# Functional Assessment of the Hautmann Ileal Neobladder with Chimney Modification Using Uroflowmetry and a Questionnaire

**DOI:** 10.1155/2016/8209589

**Published:** 2016-11-29

**Authors:** Yong Seong Lee, Ha Bum Jung, Don Kyoung Choi, Sung Tae Cho, Ki Kyung Kim, Young Goo Lee

**Affiliations:** Department of Urology, Hallym University College of Medicine, Seoul, Republic of Korea

## Abstract

Urinary diversion reconstruction is essential after radical cystectomy and neobladder reconstruction is accepted as a fine option. This study included 51 patients, who underwent radical cystectomy with orthotopic neobladder reconstruction by a Hautmann ileal neobladder with chimney modification from 2006 to 2014. Functional outcomes were evaluated using a questionnaire and uroflowmetry. Perioperative complications were analyzed retrospectively. The mean follow-up period was 36.1 months. Eighty-six percent of patients voided without clean intermittent catheterization (CIC) assistance. CIC was used 1-2x per day or every time they voided in 8% and 6% of patients, respectively, and 71% of patients were continent. The percentages of patients who used 1, 2, 3-4, and ≥5 pads per day were 15%, 6%, 2%, and 6%, respectively. Daytime and nighttime continence were achieved in 86% and 69% of patients, respectively. Daily mucus leakage was reported in 69% of patients. The mean maximum neobladder capacity, voided volume, postvoid residual volume, and maximum flow rate were 413.2 mL, 370.6 mL, 43.7 mL, and 20.8 mL/s, respectively. Eighteen early and 5 late complications developed in 13 and 5 patients, respectively. Reoperations were needed in 7 patients. The Hautmann ileal neobladder with chimney modification provided satisfactory results regarding functional outcomes.

## 1. Introduction

Until recently, radical cystectomy with urinary diversion was accepted as the gold standard for muscle-invasive (T2) bladder cancer and serious T1G3 bladder cancer [1, 2]. Since the 1980s, various types of neobladder (NB) reconstruction procedures have been developed, and the orthotopic NB is currently a common method for urinary diversion after radical cystectomy. However, there is the possibility of voiding problems following NB reconstruction, which makes patients hesitant about this surgery even though a NB provides cosmetic advantages, high quality of life, and psychological benefits [3]. Surgeons and patients need to have reasonable expectations of the functional outcomes following this surgery [4].

Several publications have reported on the functional outcomes of different techniques of NB reconstruction [5–10]. For better voiding function, NB reconstruction should aim to obtain a high capacity, low pressure, and easily emptied continent reservoir. Therefore, most previous studies evaluated the function of NBs using uroflowmetry or urodynamic tests. However, voiding status cannot be completely expressed with only objective measures such as uroflowmetry or urodynamic tests. Actually, many guidelines recommend that physicians use a questionnaire while evaluating voiding function because assessment of subjective symptoms is essential [11, 12]. As far as we know, few studies have reported the functional outcomes of NBs using a questionnaire. Additionally, functional outcomes of Hautmann ileal NBs have not been reported.

In this study, we evaluated detailed functional outcomes using uroflowmetry and a questionnaire assessing the Hautmann ileal NBs with chimney modification.

## 2. Materials and Methods

### 2.1. Patient Population

Between April 2006 and June 2014, 51 patients (41 men and 10 women) who underwent radical cystectomy and orthotopic NB reconstruction were enrolled in this study. All of the procedures were performed by a single surgeon. In our institution, orthotopic NBs were only offered to patients without contraindications [13]. The data from patient medical records were analyzed retrospectively and based on results obtained from each patient ≥ 12 months after surgery. The mean (range) age was 61.2 years (45–79). All patients were able to void without assistance prior to surgery. Simultaneous adjuvant chemotherapy or nephroureterectomy was received by 4 patients (8%) or 1 patient (2%), respectively. All patients had transitional cell carcinoma and 2 patients (4%) had positive lymph nodes according to their pathology reports. The mean (range) follow-up period was 36.1 (12–65) months. All study procedures were approved by the Institutional Review Board at Hallym University College of Medicine.

### 2.2. Surgical Technique

**Figure 1:**
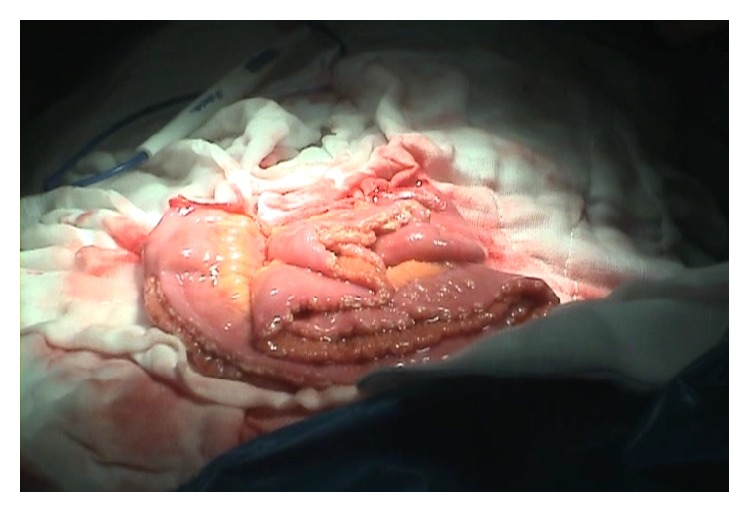
Opened bowel segment along the antimesenteric border.

**Figure 2:**
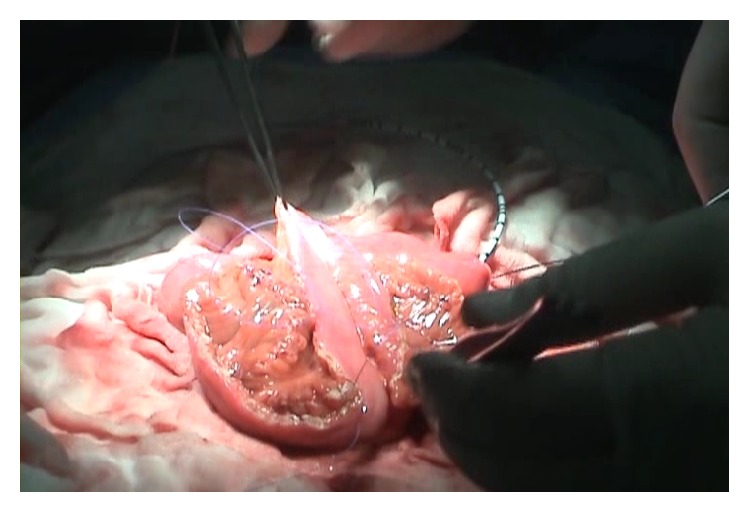
The detubularized 60 cm of the ileal segment folded into a W shape.

Pelvic lymphadenectomy was performed during every radical cystectomy. NB reconstruction was performed according to the technique of the Hautmann ileal NB with chimney modification, as described previously [14]. Approximately 15–20 cm from the ileocecal valve, a 60–70 cm ileal segment was isolated. The most proximal 10–15 cm of the isolated ileal segment (chimney) was not detubularized. The remaining 55 cm of the bowel was opened along the antimesenteric border (Figure 1). The detubularized 60 cm of the ileal segment was then folded into a W shape (Figure 2). The back wall of the W was oversewn with a running and locking technique using an absorbable suture. Both ureters were anastomosed to the anterolateral wall of the chimney. Prior to this procedure, French gauge size 5 (5 Fr) ureteral stents were inserted. The pouch was closed by sewing the outer walls together, starting at the most caudal portion and leaving the remainder of the urethral anastomosis site open. Urethral anastomosis was performed by placing a 20 Fr silicone urethral catheter into the urethra. Postoperatively, 50 cc of saline irrigation was performed every 6 h for the first week to prevent mucosal plug formation and obstruction. The ureteral stents were removed 2 weeks after surgery and retrograde pyelography was performed to determine if anastomotic leakage or strictures were present. The urethral catheter was removed 15 days after surgery. After removal of the urethral catheter, the patients were educated on how to void every 4–6 h using the Valsalva maneuver.

### 2.3. Postoperative Follow-Up

Patients underwent follow-up evaluations for 2 months after surgery and then every 3 months for 2 years. During the third and fourth years, the patients were evaluated every 6 months and were evaluated annually thereafter. At follow-up evaluations, physical examinations were performed. In addition, the routine follow-up tests included laboratory tests, urinary cytology, chest X-rays, and computed tomography. Cystourethrography was performed on patients who developed urinary retention. In this study, all complications were classified as early (≤3 months after surgery) or late (>3 months).

Voiding patterns and continence status were evaluated using a questionnaire. The questionnaires were completed by each patient 12 months after surgery. Patients were considered continent if they were able to remain dry without using protection or if they remained dry by voiding at regular intervals during the daytime. Clean intermittent catheterization (CIC) was recommended for patients with a postvoid residual volume (PVR) of >150 mL. Uroflowmetry was performed to assess NB capacity, postvoid residual volume, and urinary flow rate 12 months after surgery, which is the minimum period that the bowel needs for its adaptation to a new role [4].

## 3. Results

The functional outcomes are presented in Table 1. Spontaneous voiding without CIC assistance occurred in 44 patients (86%), while 4 patients (8%) voided with CIC assistance once or twice per day and three patients (6%) could not void spontaneously and depended completely on CIC assistance. Continence was defined as “freedom of pads.” Continence was seen in 36 patients (71%). Among the 15 patients who had urinary incontinence, 8 patients (15%) used 1 pad per day, 3 patients (6%) used 2 pads, and 1 patient (2%) used 3-4 pads. The other 3 patients (6%) had severe urinary incontinence and used 5≥ pads per 24 h. Among the 15 patients who used pads, 8 (15%) wore pads only at night, while the other 6 (12%) used pads during both the day and the night. None of the patients used pads only during the day. Daytime and nighttime continence were achieved in 45 (88%) and 36 patients (71%), respectively. In addition, 29 patients (57%) voided without CIC and were continent without a pad as well. Most patients had mucus leakage; 35 patients (69%) had mucus leakage every day, 3 patients (6%) had leakage once per week, and 13 patients (25%) had no mucus leakage.

The results of uroflowmetry are listed in Table 2. Uroflowmetry was done in 44 of the 51 patients; 3 patients who depended on CIC assistance and 4 patients with severe urinary incontinence were not able to undergo uroflowmetry. The mean (range) maximum NB capacity, voided volume, PVR, and maximum flow rate were 413.2 (267–695) mL, 370.6 (230–677) mL, 43.7 (9–132) mL, and 20.8 (8.1–39.0) mL/s, respectively.

The early and late complications in the 51 patients are summarized in Table 3. There were 18 early complications in 13 patients (25%, 10 men and 3 women) and 5 late complications in 5 patients (10%, 4 men and 1 woman). The most common early complications were ileus (15%) and wound infection (12%). The most common late complications were ureteroneobladder stricture (4%) and urethra-neobladder stricture (4%). In total, 7 complications required reoperation; all seven complications were solved after reoperation and did not affect the voiding functions of the NBs.

## 4. Discussion

Urinary diversion reconstruction is essential after radical cystectomy, which is still accepted as the best treatment for muscle-invasive and some superficial high-grade bladder cancers. The gold standard for urinary diversion is an ileal conduit, because most urologists and patients primarily focus on cancer eradication [15]. Recently, the NB has been accepted as another suitable option for urinary diversion due to its cosmetic advantages, high quality of life, and psychological benefits. The choice between the different types of urinary diversion is often based on an individual assessment considering the risks, benefits, and lifestyle of the patient, without compromising the primary surgical goals [16]. Because improvements in surgical techniques have led to low mortality and complication rates over the past few decades, several NB reconstruction techniques have been developed that achieve better functional outcomes and have a lasting impact on the quality of life in patients [15].

In 1997, Lipper and Theodorescu first described the procedure of NB reconstruction [17]. In 2000, Hollowell et al. demonstrated that the technique of the Hautmann ileal NB with chimney modification is safe and feasible and has favorable surgical outcomes compared to other techniques [14]. As presented in this paper, there are several advantages of the chimney modification. The technique is relatively easy to perform and creates a reliable ureterointestinal anastomosis without tension. In addition, the ureters do not compete with the bowel mesentery and are therefore less at risk of angulation and subsequent obstruction in cases of NB overdistention. Moreover, if reexploration is needed, it allows for easier identification and revision of the ureterointestinal anastomosis than the classic Hautmann technique. In spite of these advantages, few studies have reported on the functional outcomes of the Hautmann ileal NB with chimney modification.

Voiding function of the NB is the factor that has the strongest influence on the quality of life and satisfaction in patients. Until recently, functional outcomes of orthotopic NBs of various types have been reported. Marim et al. evaluated the functional results of 20 orthotopic “W” ileal NBs, and the mean NB capacity and PVR were 584.7 mL and 83.5 mL, respectively. In addition, 85% of patients had complete daytime incontinence [6]. In another study, Miyake et al. reported that the mean maximum flow rate, voided volume, and postvoid residual volume of 82 orthotopic sigmoid NBs were 18.6 mL/s, 345.3 mL, and 24.5 mL, respectively [7]. Zhong et al. reported that the mean maximum flow rate, voided volume, and postvoid residual volume of 56 “U-shaped” ileal NBs were 16.2 mL/s, 317.9 mL, and 22.7 mL, respectively [10]. Most studies report daytime and nocturnal continence rates of 87–96% and 57–86%, respectively [6–8, 10].

In most previous studies, uroflowmetry or urodynamic tests were used as the main method of evaluating functional outcome of various NBs. However, these objective measures may be insufficient to express voiding symptoms of patients with NBs, and a difference can exist between the objective measures and the voiding symptoms and related quality of life of patients. In an effort to determine more detailed functional outcomes, we used a questionnaire to assess voiding status. In 2004, Avery et al. presented an ICIQ questionnaire that is a brief, reliable, and worldwide measure for evaluating urinary incontinence. In the report, the amount of leaked urine and frequency of urinary leakage are considered main scoring factors because these factors can significantly affect quality of life [18]. Another study reported a systemic scoring system showed that those who rely on CIC exhibit a significantly reduced quality of life in all health domains, in patients who acquired neurogenic bladder secondary to spinal cord injury [19]. Patients with NBs who should depend on CIC right after radical cystectomy can suffer similar negative impact on quality of life. In addition to whether the patients use CIC or not, it can easily be assumed that the frequency of catheterization may affect their quality of life. Thus, our questionnaire contains questions about the number of pads used, frequency of CIC assistance, and amount of mucus leakage.

As a result of our study, 86 percent of patients voided spontaneously without CIC assistance; this is similar to or better than the results described in other studies (67–91%). Daytime and nighttime continence rates were 88% and 71%, respectively, which were comparable to those reported in other studies (87–93% and 57–86%, resp.) [6–8, 10]. Lower nighttime continence rates presumably result from the absence of the sphincter-detrusor reflux, decreased rhabdosphincter tone, and increased diuresis at night; this theory supports our results indicating that none of the patients used pads only during the day [20]. Pads were not used by 71% of patients and 15% of patients only used one pad per day. Daily mucus leakage was reported by 35 patients (69%); most continent patients reported that they leaked mucus several times a day. Conversely, the more severe the incontinence was, the less mucus that the patient leaked. This may be because the period of time that urine remains in the NB is a factor that relates to mucus leakage.

Uroflowmetry has been used to evaluate the voiding function of orthotopic NBs in many studies. The mean maximum NB capacity, PVR, and maximum flow rate were 413.2 mL, 43.7 mL, and 20.8 mL/s, respectively, as listed in Table 2. According to previous studies, the mean maximum NB capacity, PVR, and maximum flow rate were 240.4–628.9 mL, 22.7–91.0 mL, and 11.2–18.6 mL/s, respectively, which are similar to the results of this study [6–8, 10]. We attempted to evaluate the uroflowmetry patterns using the previously presented classifications. However, most patients had the fractionated or staccato type of pattern because they voided with several Valsalva maneuvers; only 8 rough Bell types were observed.

Many reports have evaluated the complication rates of other types of orthotopic NBs to verify the safety of NB reconstruction. Recent studies report that 22–34% and 13–26% of patients had early and late complications, respectively, and 1–29% of patients had reoperation for postoperative complications [5, 9, 10]. In our study, 25% and 10% of patients with the Hautmann NB had early and late complications, respectively, and 14% of patients needed reoperations. These results are comparable with those in previous studies reporting other types of NBs. Although these results show acceptable functional outcomes and complication rates in Hautmann NB reconstruction, the number of patients evaluated in this study was small, which decreases the statistical reliability. We evaluated the functional outcomes and complications of orthotopic NBs; however, we could not identify the factors affecting the results. Selection bias may have been introduced because the data were analyzed retrospectively. However, the detailed voiding functional results of NBs may be informative to patients who need to decide on the type of urinary diversion before radical cystectomy. Further, large, prospective, and long-term studies with the tools to demonstrate the functional outcomes are needed in order to draw a realistic conclusion.

## 5. Conclusions

We evaluated the functional outcomes of Hautmann ileal NBs with a detailed questionnaire and uroflowmetry examination. Considering the results of the present study, Hautmann ileal NBs could be recommended for patients who need a radical cystectomy.

## Questionnaire for Patients with Neobladder

This questionnaire is designed to measure quality of life issues in patients with bladder cancer and/or urinary diversions. In order to help us get the most accurate assessment, it is important that you answer all questions honestly and completely. As with all medical records,* information contained within this survey will remain strictly confidential*.

1. Do you catheterize your neobladder?
  Yes
  No
2. Over the past 4 weeks, how often did you catheterize your neobladder each day?
  1-2 times per day
  3-4 times per day
  More than 4 times per day
  I always have to catheterize to empty my neobladder.
3. Do you use pads?
  Yes
  No
4. How many pads per day do you typically use?
  1-2 pads per day
  3-4 pads per day
  More than 5 pads per day
5. When do you use pads?
  Day only
  Night only
  Day and night
6. Do you leak mucus?
  Yes
  No
7. How often do you leak mucus?
  Every day
  About once a week
  Less than once a week
  Not at all

## Figures and Tables

**Table 1:** Voiding patterns of Hautmann ileal neobladder with chimney modification.

CIC frequency	
0	44 (86)
1-2	4 (8)
3-4	0 (0)
Always	3 (6)
Number of pads/24 hrs	
0	36 (71)
1	8 (15)
2	3 (6)
3-4	1 (2)
≥5	3 (6)
Pad use time	
Day only	0 (0)
Night only	8 (15)
Day/night	7 (14)
Daytime/nighttime continence	
Daytime continence	45 (88)
Nighttime continence	36 (71)
Pad wetness	
Almost dry	9/15 (18)
Slightly wet	2/15 (4)
Wet	3/15 (6)
Soaked	1/15 (2)
Mucus leakage	
Everyday	35 (69)
Once per week	3 (6)
Not at all	13 (25)

**Table 2:** The voiding function of Hautmann ileal neobladder with chimney modification.

Uroflowmetry parameters (*n* = 44)	
Maximum neobladder capacity, mL	413.2 (267–695)
Voided volume, mL	370.6 (230–677)
Postvoid urinary volume, mL	43.7 (9–132)
Maximum flow rate, mL/s	20.8 (8.1–39.0)

**Table 3:** Early and late complications in patients with Hautmann ileal neobladder with chimney modification.

Complications	Total	*n* (%) patients
		Requiring re-op
Early		
Ileus	8 (15)	1 (2)
Wound infection	6 (12)	
Pyelonephritis	2 (4)	
Persistent urine leak	1 (2)	
Vagino-neobladder fistula	1 (2)	1 (2)
Total/patients	18/13 (25)	2/2 (4)
Late		
Ureteroneobladder stricture	2 (4)	2 (4)
Urethra-neobladder stricture	2 (4)	2 (4)
Neobladder stone	1 (2)	1 (2)
Total/patients	5/5 (10)	5/5 (10)
